# Supplementary material for: Causal Relationship between Aging and Anorexia Nervosa: A White-Matter-Microstructure-Mediated Mendelian Randomization Analysis
Source: Biomedicines. 2024 Aug 16;12(8):1874. doi: 10.3390/biomedicines12081874 (PMC11351342; doi:10.3390/biomedicines12081874)
Supplement: Supplementary file 1 [file biomedicines-12-01874-s001.zip › Supplementary Figure/Figure S4/Singe SNP plot of MR analysis for TL to ubm-b-1771.pdf]

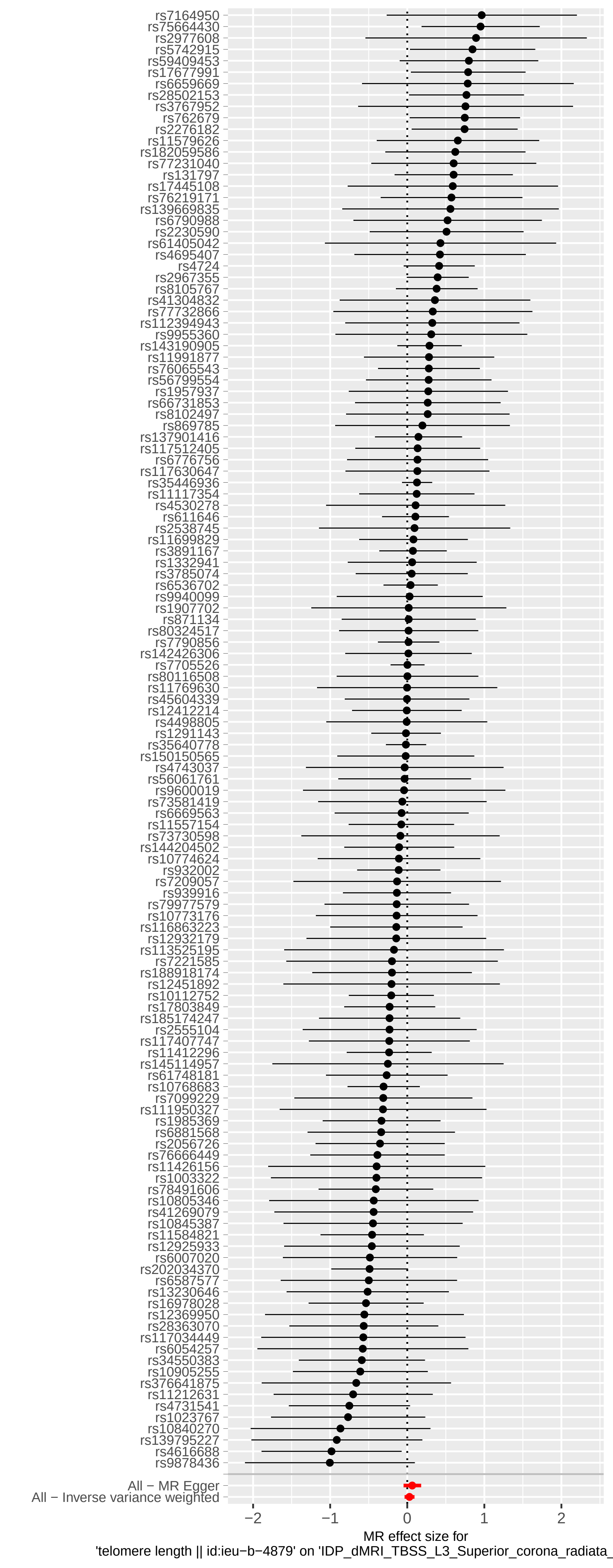
rs7164950
rs75664430
rs2977608
rs5742915
rs59409453
rs17677991
rs6659669
rs28502153
rs3767952
rs762679
rs2276182
rs11579626
rs182059586
rs77231040
rs131797
rs17445108
rs76219171
rs139669835
rs6790988
rs2230590
rs61405042
rs4695407
rs4724
rs2967355
rs8105767
rs41304832
rs77732866
rs112394943
rs9955360
rs143190905
rs11991877
rs76065543
rs56799554
rs1957937
rs66731853
rs8102497
rs869785
rs137901416
rs117512405
rs6776756
rs117630647
rs35446936
rs11117354
rs4530278
rs611646
rs2538745
rs11699829
rs3891167
rs1332941
rs3785074
rs6536702
rs9940099
rs1907702
rs871134
rs80324517
rs7790856
rs142426306
rs7705526
rs80116508
rs11769630
rs45604339
rs12412214
rs4498805
rs1291143
rs35640778
rs150150565
rs4743037
rs56061761
rs9600019
rs73581419
rs6669563
rs11557154
rs73730598
rs144204502
rs10774624
rs932002
rs7209057
rs939916
rs79977579
rs10773176
rs116863223
rs12932179
rs113525195
rs7221585
rs188918174
rs12451892
rs10112752
rs17803849
rs185174247
rs2555104
rs117407747
rs11412296
rs145114957
rs61748181
rs10768683
rs7099229
rs111950327
rs1985369
rs6881568
rs2056726
rs76666449
rs11426156
rs1003322
rs78491606
rs10805346
rs41269079
rs10845387
rs11584821
rs12925933
rs6007020
rs202034370
rs6587577
rs13230646
rs16978028
rs12369950
rs28363070
rs117034449
rs6054257
rs34550383
rs10905255
rs376641875
rs11212631
rs4731541
rs1023767
rs10840270
rs139795227
rs4616688
rs9878436
All − MR Egger
All − Inverse variance weighted
−2
−1
0
1
2
MR effect size for
'telomere length || id:ieu−b−4879' on 'IDP_dMRI_TBSS_L3_Superior_corona_radiata_
